# Editorial: Plant growth-promoting rhizobacteria (PGPR) and plant hormones: an approach for plant abiotic stress management and sustainable agriculture

**DOI:** 10.3389/fmicb.2023.1285756

**Published:** 2023-09-19

**Authors:** Kunal, Kumar Pranaw, Kailash Chand Kumawat, Vijay Singh Meena

**Affiliations:** ^1^Department of Microbiology, Faculty of Allied Health Sciences, SGT University, Gurugram, Haryana, India; ^2^Department of Environmental Microbiology and Biotechnology, Faculty of Biology, Institute of Microbiology, University of Warsaw, Warsaw, Poland; ^3^Department of Industrial Microbiology, Jacob Institute of Biotechnology and Bioengineering (JIBB), Sam Higginbottom University of Agriculture, Technology and Sciences (SHUATS), Prayagraj, Uttar Pradesh, India; ^4^CIMMYT-Borlaug Institute for South Asia (BISA) Pusa, Samastipur, Bihar, India

**Keywords:** drought tolerance, halophiles, heavy metal stress, mineral solubilization, phytohormones

The increasing threats of climate change exert abiotic stresses including high/low temperatures, salinity, heavy metals, drought, and floods, on crops, thereby affecting crop productivity, and nutrient uptake. Combating the consequences of climate change is critical for meeting the food requirements of the growing global population. Plant growth-promoting rhizobacteria (PGPR) are a vital group of beneficial soil bacteria involved in a range of ecologically significant activities. They promote plant growth by alleviating abiotic stress, supporting nutrient uptake, and increasing crop productivity through various mechanisms, particularly involving plant hormones and metabolites. This Research Topic has been meticulously organized and features six original research and review articles.

PGPR phytohormones and rhizobacterial hormonal communication govern a plant's response to abiotic stress. Aloo et al. reviewed how PGPR alleviate plant stress through both direct mechanisms (increased nutrient availability, mineral acquisition, water absorption, development of exopolysaccharides, biofilm formation, and secretion of organic solutes, such as sugars, organic acids, amino acids, and polyamines) and indirect mechanisms [chemotaxis, phytohormone synthesis, activation of antioxidant defense systems, 1-aminocyclopropane-1-carboxylate (ACC) deaminase activity, and the regulation of stress-responsive genes]. Furthermore, PGPR reduces heavy-metal-induced plant stress by producing siderophores, low- molecular-weight chelators that bind to heavy metals like cadmium, iron, lead, zinc, etc., to form complexes.

Beneficial soil microbes, such as PGPRs capable of promoting plant growth under stress conditions, are often isolated from harsh environments and are known as extremophiles. However, the challenge lies in identifying and studying the potential functional roles of several non-culturable PGPRs. Marghoob et al. reported the utilization of metagenomics (sequencing the total microbial content of the soil) to characterize the diversity of bacteria, fungi, and archaea in saline and heavy metal-stressed soils. Dominant microbial communities include *Euryarchaeota* (archaea)*, Actinobacteria, Proteobacteria, Planctomycetota, Firimicutes, Patescibacteria* and *Acidobacteria* (bacteria), and *Ascomycota* (fungi). Both salinity and heavy metals alter microbial community diversity, potentially affecting plant growth positively or negatively. Such alterations influence the potential functional capabilities of microbial communities, including nutrient cycling, pathogen suppression, and plant stress tolerance. These changes could have adverse effects on both crop and human health.

Halophiles and halo-tolerant PGPR isolates are extremophiles that exhibit high tolerance to saline conditions. These PGPRs enhance plant growth by establishing interactions with plants, allowing them to thrive in nutrient-deficient environments. Oliva et al. studied the effect of the PGPR bacterial consortium (*Staphylococcus succinus* + *Bacillus stratosphericus*) on the growth of maize seedlings under saline stress. Seed treatment with PGPR bacterial consortium improves plant growth and partially restores the root architecture system under saline stress conditions compared to the uninoculated control. The production of indole acetic acid (IAA) and ACC deaminase by the consortium reduces ethylene stress, thereby improving root development and plant growth. The authors suggest that this is an effective strategy to enhance sustainable agriculture in saline soils. Additionally, soil salinity impacts nutrient availability for plants. Marghoob et al. reported a deficiency of sodium- induced potassium (K) in saline-affected soils, which is essential for plant survival. In the study, a consortium of five K-solubilizing bacterial isolates (*Enterobacter hormaechei, Citrobacter braakii, Pseudomonas putida, Erwinia iniecta, Pantoea agglomerans*) was inoculated in rice grown under saline-sodic conditions to assess K assimilation and yield improvement. A significant increase in K uptake and the K/Na ratio in roots and shoots signifies the potential role of K-solubilizing bacteria in increasing grain yield and biomass.

The role of halotolerant bacterial isolates in mitigating saline and drought stress simultaneously is also an interesting study conducted by Nawaz et al. in fenugreek (*Trigonella foenumgraecum* L.) crops. The *Bacillus subtilis* ESR-08 strain was isolated from the halotolerant plant *Fagonia cretica* in the Little Rann of Kachchh region of India. The selected isolate, exhibiting plant growth-promoting characteristics, was used to treat fenugreek seeds to study plant growth and development under greenhouse conditions. The secretion of IAA, gibberellins, and the production of hydrogen cyanide, siderophores, exopolysaccharides (EPS), ammonia, cellulase, protease, pectinase, and chitinase by the isolate help in tolerating biotic and abiotic stress in plants. Under *in-vitro* conditions, a 56% reduction in *Fusarium oxysporum* growth by *Bacillus subtilis* ESR-08 strain was observed, along with good growth under salinity (4 and 6 dSm-1), pH 5-9, drought (10–30% PEG 6000), and temperatures (25–55°C), demonstrating the mitigation of biotic and abiotic stress conditions.

Bacterial signaling compounds, such as phytohormones, also help alleviate abiotic stress in plants. Subramanian et al. studied the response of Lipo-chitooligosaccharide (LCO) and thuricin 17 (Th17) phytohormones in alleviating drought stress in *Arabidopsis thaliana* plants. The exposure of drought-stressed plants to LCO showed an increase in abscisic acid (ABA) and salicylic acid (SA) content, while plants exposed to Th17 exhibited an increase in IAA and SA content. Free proline content and drought-tolerant specific proteins (nitrite reductase and thioredoxin reductase) increased in both treatments. It is concluded that global climate change is causing an increase in large-scale droughts, but the use of bacterial signaling compounds could play a significant role in mitigating the adverse effects of drought on agricultural production.

Overall, this Research Topic offers highly significant insights into the roles of various metabolites, hormones, and enzymes in modulating abiotic stress tolerance, as well as the identification of putative stress-responsive genes to enhance the tolerance of agricultural crops. Further efforts are needed to promote these services within this agroecosystem, and developing and applying them, as inoculants would be helpful for improving crop productivity.

## Author contributions

K: Writing—original draft. KP: Writing—review and editing. KK: Writing—review and editing. VM: Writing—review and editing.

